# Dose‐Dependent Cannabidiol‐Induced Elevation of Intracellular Calcium and Apoptosis in Human Articular Chondrocytes

**DOI:** 10.1002/jor.24430

**Published:** 2019-08-26

**Authors:** Martina Winklmayr, Martin Gaisberger, Michael Kittl, Julia Fuchs, Markus Ritter, Martin Jakab

**Affiliations:** ^1^ Institute of Physiology and Pathophysiology Paracelsus Medical University 5020 Salzburg Austria; ^2^ Ludwig Boltzmann Institute for Arthritis and Rehabilitation Paracelsus Medical University 5020 Salzburg Austria; ^3^ Gastein Research Institute Paracelsus Medical University 5020 Salzburg Austria

**Keywords:** osteoarthritis, cannabidiol, CBD, chondrocyte, apoptosis, calcium, Ca^2+^, viability

## Abstract

Cannabidiol (CBD) is the most abundant non‐psychoactive compound of *Cannabis sativa* extracts. Cannabinoids have been shown to exhibit anti‐inflammatory, analgesic, antioxidant, neuroprotective, and anti‐tumorigenic effects. In the present study, we investigated the effects of CBD on human articular chondrocytes. Cell viability was determined by Resazurin assays. Apoptosis was analyzed by annexin‐V/7‐actinomycin D (7‐AAD) staining followed by flow cytometry. Caspase 3/7 activity was measured with caspase assays. Intracellular Ca^2+^ ([Ca^2+^]*_i_*) was monitored by time‐lapse fluorescence imaging. The perforated whole‐cell patch‐clamp technique was used for measuring the cell membrane potential. Erk1/2 phosphorylation was assessed by western blot analysis. The chondrocyte cell line C28/I2 and primary chondrocytes showed a reduced viability after treatment with concentrations of CBD greater than 4 µM. This apoptotic effect was accompanied by an increase of caspase 3/7 activity and an increase in the early apoptotic cell population. CBD elevated [Ca^2+^]*_i_*, which was accompanied by depolarization of the cell membrane potential. The increase of [Ca^2+^]*_i_* was abrogated, when Ca^2+^ was omitted from the bath solution, indicating an influx of extracellular Ca^2+^. The cannabinoid receptor 1 (CB1) antagonist AM251 inhibited the Ca^2+^ influx triggered by CBD. Preincubation with AM251 reduced the toxic effects of CBD. By looking for mediators of the apoptotic CBD effect downstream of the CB1 receptor, enhanced Erk1/2 phosphorylation could be detected after CBD treatment. However, this Erk1/2 activation proved to be unaffected by CB1 receptor blockage. The present study demonstrates that CBD promotes apoptosis and [Ca^2+^]*_i_* elevation in human articular chondrocytes via a CB1‐receptor‐mediated mechanism. © 2019 The Authors. *Journal of Orthopaedic Research* ^®^ published by Wiley Periodicals, Inc. on behalf of Orthopaedic Research Society J Orthop Res 37:2540–2549, 2019

Osteoarthritis (OA) is a leading cause of chronic disability. Progressive cartilage damage, inflammation of the synovial compartment, subchondral bone alterations, and osteophyte formation characterize this disease. Elevated inflammatory and catabolic responses finally lead to a loss of joint architecture and deformity.^1^ Currently, there are no disease‐modifying OA drugs available and treatment is limited to pain reduction, improvement of joint mobility and functionality and delay of disease progression. Often in severe cases, it ultimately results in joint replacement. OA is a major public health problem among the increasing aged and obese population, therefore the development and investigation of new therapeutics, which hinder its progression, is a major focus of OA research.

Endocannabinoids (ECs), cannabinoids derived from the cannabis plant *Cannabis sativa* and synthetic cannabinoid analogs have been attributed anti‐inflammatory, antitumorigenic, analgesic, and psychoactive effects.^2^, ^3^ ECs, cannabinoids and their derivatives exert their actions through a variety of receptors: Cannabinoid receptors 1 and 2 (CB1, CB2) are the primary targets of the main endogenous ligands anandamide (AEA) and 2‐arachidonylglycerol (2‐AG), but cannabinoids have also been shown to interact with the transient receptor potential cation channel subfamily V member 1 (TRPV‐1), G protein‐coupled receptors GPCR18, and GPCR55, peroxisome proliferator‐activated receptor γ (PPARγ), and other targets.^4^, ^5^, ^6^

In recent years, an increasing interest in the EC system as a target for therapeutic treatment of joint diseases has emerged.^7^, ^8^ The EC system has been shown to act as a modulator of OA pain manifestations as well as emotional and stress‐related responses produced by OA pain in animal models. Induced OA pain led to an increase in plasma levels of 2‐AG in mice,^9^, ^10^ whereas CB2 receptor activation attenuated the development of pain and central sensitization in an OA rat model.^11^ CB2 knockout mice have a higher susceptibility to age‐related OA and develop worse OA symptoms in induced OA compared with WT mice. The administration of the CB2‐selective agonist HU308 also protected WT mice against the development of OA, but not the CB2 knockout mice.^12^

In humans, a positive correlation between plasma 2‐AG levels and knee pain scores could be demonstrated^9^ as well as a negative correlation between joint chondropathy scores and spinal cord CB2 receptor expression.^11^ Clinical studies have demonstrated that CB1 and CB2 receptor messenger RNA and proteins are expressed in the synovia of OA and rheumatoid arthritis patients and that the ECs 2‐AG and AEA were present in the synovial fluid of these patients, but not in the synovia of individuals without joint symptoms.^13^ Cannabinoid receptors are expressed in human OA cartilage, human primary chondrocytes and in osteocytes of the underlying bone.^14^

The influence of cannabinoids on chondrocyte metabolism and matrix formation has been investigated in a couple of studies. Human primary chondrocytes obtained from OA patients and treated with the pro‐inflammatory cytokine interleukin‐1β expressed fewer matrix‐degrading metalloproteinases MMP‐3 and MMP‐13 when co‐treated with the synthetic cannabinoid WIN‐55.^15^ In another study, WIN‐55 inhibited the activity of disintegrin and metalloproteinase with thrombospondin motifs 4 (ADAMTS‐4), an enzyme important in cartilage breakdown by degradation of aggrecan, the main proteoglycan of the cartilage matrix.^16^ The cannabinoid agonizts HU‐210 and WIN‐55 were tested on cartilage breakdown in bovine nasal cartilage explant cultures and showed inhibition of interleukin‐1β induced matrix degradation.^17^ In contrast to the above‐mentioned studies, which suggest a protective role of cannabinoids in cartilage metabolism, the results of Gómez et al.^18^ indicate the pro‐apoptotic effect of the endocannabinoid AEA in a murine chondrogenic cell line and immortalized human chondrocyte cell lines. In addition to ECs and synthetic cannabinoids cannabidiol (CBD), a major non‐psychoactive component of *Cannabis* extracts has been shown to have anti‐arthritic potency in murine collagen‐induced arthritis^19^ and to reduce inflammation and pain in a rat model of arthritis when applied transdermally.^20^ In the present study, we investigated the effects of CBD on the cell viability and Ca^2+^ homeostasis in human articular chondrocytes.

## MATERIALS AND METHODS

### Reagents

If not otherwise noted, chemicals were obtained from Sigma‐Aldrich (Darmstadt, Germany). AM251, AM630, were obtained from Tocris Bioscience (Abingdon, UK), CBD from Trigal Pharma GmbH (Vienna, Austria), 1,2‐bis(o‐aminophenoxy)ethane‐*N*,*N*,*N*′,*N*′‐tetraacetic acid (BAPTA)/AM from Thermo Fisher Scientific (Darmstadt, Germany). Histamine and CdCl_2_ were dissolved in water, CBD and Nifedipine in ethanol, all others in dimethylsulfoxide. To exclude solvent effects, all solvents were tested at the highest applied concentrations prior to all experiments.

### Cell Culture

Human immortalized C28/I2 cells, originating from cells isolated from rib cartilage,^21^ were cultured in Dulbecco's modified Eagle's medium (DMEM)/HAM's F‐12 medium (Biochrom, Darmstadt, Germany) supplemented with 5 % fetal bovine serum (FBS Superior; Biochrom) and antibiotic‐antimycotic solution (100 U/ml penicillin, 0.1 mg/ml streptomycin, 0.25 µg/ml amphotericin‐B; Sigma‐Aldrich) in a humidified atmosphere at 37 °C and 5 % CO_2_.

Human primary chondrocytes were isolated from total knee arthroplasty samples with informed consent and ethical approval by the Ethics Committee of Salzburg (415‐E/1965/4‐2015). All patients were female and all samples were macroscopically graded between 3 and 4 or 4 (Outerbridge classification). Cartilage samples of the whole tibial area and femur condyles were removed from subchondral bone, minced, washed in phosphate‐buffered saline (PBS) and digested overnight in DMEM/HAM's F‐12 supplemented with FBS and 2 mg/ml collagenase Type II (275 units/mg; Thermo Fisher Scientific) at 37 °C on a shaker. After 24 h, the cells were centrifuged, washed twice with PBS, resuspended in medium and seeded as required for the following experiments. For 7‐hydroxy‐3H‐phenoxazin‐3‐one‐10‐oxide sodium salt (Resazurin) assays and Fura‐2 assays, cells were always used at the first passage.

### Cell Viability Assay/Resazurin Assay

Ten thousand cells per well were seeded into transparent 96‐well microplates (CytoOne; Starlab, Hamburg, Germany) and grown overnight. After 24 h, the medium was replaced by the medium without FBS (−FBS) and grown for another 24 h before treatment. Cells were then incubated with CBD in medium −FBS at concentrations as indicated in the individual experiments. Samples were incubated at 37 °C for 2 or 24 h and then the supernatants were replaced by 100 µl medium −FBS containing 0.5 mM Resazurin. After 1 h of incubation, supernatants were transferred to a new 96‐well plate and stored at −20 °C until measurement. Fluorescence was measured in a Spark multimode reader (Tecan, Grödig, Austria). Blank well values (medium only) were subtracted and viability was related to untreated cells. For BAPTA/ethylenediaminetetraacetic acid (EDTA) or blocker experiments, treatment was for 2 or 5 h, respectively.

### Caspase 3/7 Activity

Cells were seeded like in the Resazurin assay. Five hours post‐treatment (50 µl total volume in medium −FBS), 50 µl of Caspase‐Glo 3/7 assay substrate (Promega, Mannheim, Germany) were added and the assay performed according to the manufacturer's instructions. Luminescence was measured in a Spark multimode reader (Tecan). Independent experiments were measured in duplicates.

### Annexin‐V/7‐actinomycin D (7‐AAD) Staining

For assessment of phosphatidylserine exposure at the cell surface by annexin‐V binding and cell membrane integrity by 7‐AAD staining, cells were seeded in 35 mm diameter Petri dishes at a density of 300,000 cells/dish. Following overnight culture under standard conditions, the medium was changed to −FBS. The following day, cells were either left untreated or incubated with varying concentrations of CBD for 5 h. Thereafter, the cells were harvested and the annexin‐V/7‐AAD assay was performed following the manufacturer's protocol (BioLegend, San Diego, CA, USA) and analyzed by flow cytometry (Cell Lab Quanta SC; Beckman Coulter, Krefeld, Germany) using the Kaluza Analysis 1.3 software (Beckman Coulter).

### Fura‐2 Assays

Intracellular Ca^2+^ was monitored by time‐lapse fluorescence imaging. Cells were grown for 24 h in 35 mm glass‐bottom Petri dishes (MatTek Corporation, Ashland, MA, USA) under standard cell culture conditions were loaded with 5 µM Fura‐2/AM plus 0.08 % Pluronic F‐127 (Molecular Probes‐LifeTechnologies, Darmstadt, Germany) for 20 min at 37 °C in the dark in medium −FBS. Cells were washed once and incubated for a further 20 min in the dark to allow for complete de‐esterification of the dye. Dishes were placed on a microscope (iMIC; TILL Photonics‐FEI, Munich, Germany) equipped with a filter set consisting of a 395 nm clean‐up filter, a 409 nm beam splitter and a 510/84 nm bandpass filter (AHF Analysentechnik, Tübingen, Germany) compatible with the Polychrome V monochromator (TILL Photonics‐FEI) used for dye excitation. Cells were alternately illuminated at 340 and 380 nm for 100–300 ms at 5‐s intervals. The emitted light was passed through the clean‐up filter, the beam splitter, and the bandpass emission filter before detection with a PCO Sensicam QE CCD camera (PCO AG, Kelheim, Germany). Life Acquisition and Offline Analysis Software (TILL Photonics‐FEI) was used for microscope, camera and monochromator control, data acquisition and analysis. Cells were continuously perfused with a solution containing (in mM): 140 NaCl, 5.6 KCl, 2.5 CaCl_2_, 1.5 MgCl_2_, 10 4‐(2‐hydroxyethyl)‐1‐piperazineethanesulfonic acid (HEPES) free acid, 4.5 d‐glucose, and 5 mannitol (pH 7.4 adjusted with NaOH, 300 mOsm/kg) at a flow rate of 2–3 ml/min. The Ca^2+^‐free extracellular solution was prepared by the omission of CaCl_2_ and addition of 2 mM ethylene glycol‐bis(β‐aminoethyl ether)‐*N*,*N*,*N*′,*N*′‐tetraacetic acid (EGTA). Drugs were added to the solution as indicated in the figure legends. The image background was recorded from cell‐free areas and subtracted from the respective signals in the regions of interest (ROIs). Data are given as the mean of maximum 340/380 nm excitation ratios detected at 510 nm.

### Patch Clamp

C28/I2 cells were seeded at a density of 300,000 cells/2 ml on 0.01 % poly‐d‐lysine (PDL) coated glass coverslips (12 mm diameter) and cultured for at least 24 h in DMEM. Coverslips were transferred to an RC‐25 recording chamber and mounted on a Nikon Eclipse inverted microscope. Patch‐clamp experiments were performed at room temperature in the perforated whole‐cell mode, which was achieved by adding 130 µM amphotericin to the pipette solution. Patch electrode resistances were 5–9 MΩ, and the recordings were started as soon as the series resistance was below 40 MΩ. After establishing the whole‐cell configuration, cells were superfused with an isotonic extracellular solution and cell membrane potentials (*V*_mem_) were recorded in the zero‐current clamp mode using an EPC‐10 amplifier controlled by PatchMaster software (HEKA, Lambrecht/Pfalz, Germany). The intracellular (pipette) solution contained (in mM): 70 K_2_SO_4_, 10 NaCl, 10 KCl, 4 MgCl_2_, 2 CaCl_2_, 5 HEPES free acid, and 10 EGTA (249 mOsm/kg, pH 7.2 adjusted with KOH). The extracellular solution contained (in mM): 140 NaCl, 5.6 KCl, 2.5 CaCl_2_, 1.5 MgCl_2_, 10 HEPES free acid, 4.5 d‐glucose, and 5 mannitol (301 mOsm/kg, pH 7.4 adjusted with NaOH). CBD was applied with the extracellular solution after reaching a stable *V*_mem_. Bath solution exchange was performed with a valve‐controlled gravity‐driven perfusion system (ALA Scientific Instruments, Farmingdale, NY, USA) at a flow rate of 3–5 ml/min. Osmolalities of intracellular and extracellular solutions were measured using a vapor pressure osmometer (Wescor, Logan, UT, USA).

### Western Blot

Cells were seeded at a density of 300,000 cells into 35 mm diameter Petri dishes, grown overnight and then switched to serum‐free for 24 h. After treatment with 15 µM CBD or additional 16 µM AM251 for 3 h, cells were lysed (cell lysis buffer 10×) + PMSF; Cell Signaling Technology, Frankfurt am Main, Germany) and sonicated. Cell lysates were mixed with sample buffer 1:1 (2× Laemmli sample buffer: 62.5 mM Tris‐HCl pH 6.8, 25 % glycerol, 2 % (w/v) SDS, 0.01 % (w/v) bromphenol blue, 0.5 % β‐mercaptoethanol) and heated for 5 min to 95 °C. Samples and standards (Precision Plus Protein WesternC Blotting Standards; BioRad, Vienna, Austria) were separated by gel electrophoresis (4–20 % Mini‐PROTEAN TGX; BioRad) and transferred to Trans‐Blot Turbo Mini Nitrocellulose Transfer Packs System (BioRad). Membranes were blocked in 5 % non‐fat dry milk (BioRad) in TBS‐T (Tris‐buffered saline pH 7.6, 0.1 % Tween‐20) for 1 h. After TBS‐T washing, the membranes were incubated overnight at 4 °C with 1:1,000 dilutions of primary antibodies (β‐Actin, p44/42 MAPK [Erk1/2], phospho‐p44/42 MAPK [Erk1/2]). The next day the membranes were washed with TBS‐T and incubated for one hour with HRP‐linked secondary antibody (1:2,000). All antibodies were purchased from Cell Signaling Technology and are listed in Table 1. After development for one minute with SignalFire ECL Reagent (Cell Signaling Technology), protein bands were visualized with the ChemiDoc MP System (BioRad). For analysis of protein expression, ImageJ (NIH, Bethesda, MD, USA) was used to calculate gray densities of target proteins in comparison to β‐actin. Gray value ratios were calculated according to the formula:

[(Phospho−p44/42MAPK(Erk1/2)/β−Actin)]/[(p44/42MAPK(Erk1/2)/β−Actin)]

**Table 1:** List of Antibodies

	Product #
Primary antibodies	
β‐Actin (13E5) rabbit mAb	#4970
p44/42 MAPK (Erk1/2)	#9102
Phospho‐p44/42 MAPK (Erk1/2) (Thr202/Tyr204)	#9101
Secondary antibody	
Anti‐rabbit IgG, HRP‐linked	#7074

HRP, horseradish peroxidase; IgG, immunoglobulin G; mAb, monoclonal antibodies; MAPK, mitogen‐activated protein kinase.

### Statistical Analysis

Statistics were performed using SPSS 24.0 software (SPSS, Chicago, IL, USA). All data are expressed as mean value ± standard deviation. Statistical analysis was performed with the Mann–Whitney *U* test or Kruskal–Wallis test for independent samples and Wilcoxon‐Rank‐Sum test or Friedman test for dependent samples. Post hoc Bonferroni correction was used for multiple comparisons. In graphs, asterisks represent *p* values of *p < 0.05; **p < 0.01; ***p < 0.001.

## RESULTS

### CBD Reduces Cell Viability and Induces Apoptosis in Human Articular Chondrocytes

**Figure 1:**
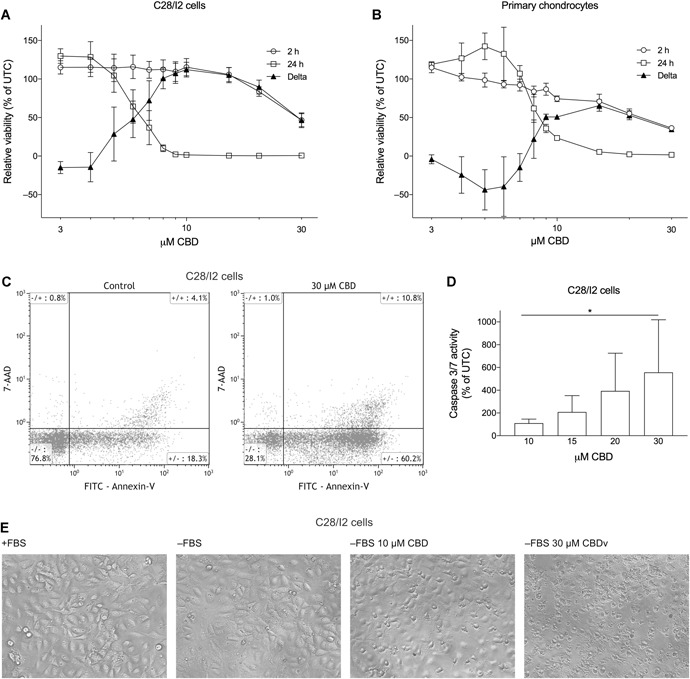
Cannabidiol (CBD) reduces viability and induces apoptosis in human chondrocytes. Relative viability of C28/I2 cells (A) or human primary chondrocytes (B) treated with 3–30 µM CBD for 2 and 24 h. Results are given as % of untreated controls (UTC). Mean ± standard deviation (SD) of three independent 7‐hydroxy‐3*H*‐phenoxazin‐3‐one‐10‐oxide sodium salt (Resazurin) experiments. (C) Flow cytometry analysis of annexin‐V/7‐actinomycin D (7‐AAD) staining in C28/I2 cells treated ± CBD. (D) Caspase 3/7 activity (% of UTC) of C28/I2 cells treated with rising concentrations of CBD. Mean ± SD of three independent experiments. (E) Microscopy images of C28/I2 cells untreated, starved, or starved and treated with 10 or 30 µM CBD for 2 h.

As a first approach, we investigated the potential cytotoxic effects of CBD on immortalized C28/I2 cells and primary human chondrocytes (passage 1). Preliminary results from experiments with CBD concentrations rising from nanomolar (1 nM) to micromolar (10 µM) had indicated a cytotoxic effect of CBD above 5 µM, but no effect on viability in the nanomolar range (Supplementary Fig. S1). After treatment with concentrations in the micromolar range (3–30 µM), cell viability was determined after 2 and 24 h of incubation with the Resazurin viability assay (Fig. 1A and B). In C28/I2 cells, the early reading (2 h) revealed a dose‐dependent decline in cell viability at concentrations above 10 µM, while concentrations below 10 µM did not seem to affect cell viability. The late reading (24 h) showed a dose‐dependent decline in cell viability already at doses above 4 µM. It has recently been shown that calculation of the difference curve of two different reading time points enables discrimination among proliferation, apoptosis and necrosis.^22^ We therefore calculated the difference curve (Delta) between the 2‐ and 24‐h reading, which shows negative values for the lowest two concentrations indicating that cells were still proliferating. The values then reached a maximum at 10 µM, indicating apoptosis with high metabolic activity at early time points and low activity at late time points. After that, the curve declines as values drop, also including the 2‐h reading (Fig. 1A). Primary chondrocytes (Fig. 1B) showed a very similar response to CBD treatment, although the decline of viability was less abrupt in the 24 h reading and the Delta curve revealed a slight shift to the right resulting in an apoptosis maximum at 15 µM. Moreover, at low CBD doses (3–7 µM), primary chondrocytes showed an enhanced proliferation at the 24‐h time point, whereas the viability of the immortalized cell line remained unchanged, possibly reflecting a higher tolerance of primary chondrocytes toward CBD at low concentrations.

Annexin‐V/7‐AAD double staining and flow cytometry analysis were performed to confirm cellular apoptosis (Fig. 1C and Table 2). After a 5‐h treatment with 30 µM CBD, there was a decline in the annexin‐V–/7‐AAD– (living) cell population (*p* = 0.0184) accompanied by a 2.6‐fold increase in the annexin‐V+/7‐AAD– (early apoptotic) population (*p* = 0.0417). Early apoptotic cells were already elevated at 15 µM CBD (*p* = 0.0015). Annexin‐V+/7‐AAD+ (late apoptotic) and annexin‐V–/7‐AAD+ (necrotic) cell populations also showed a slight increase. Apoptosis induction was additionally verified by testing caspase 3/7 activity in C28/I2 cells (Fig. 1D). After a 5‐h treatment with rising concentrations of CBD, caspase 3/7 activity was elevated compared with untreated cells (*p* = 0.0266). The induction of apoptosis and cell death was also microscopically apparent: When cells are seeded and grown for 24 h and then starved (−FBS), or starved with subsequent treatment with 10 and 30 µM CBD for 2 h, a growing number of shrunk and dead cells appeared in the CBD‐treated samples (Fig. 1E).

**Table 2:** Annexin‐V/7‐AAD Staining of C28/I2 Cells Assessed by Flow Cytometry

CBD (µM)	Annexin‐V–7‐AAD–	*p* Value	Annexin‐V+7‐AAD–	*p* Value	Annexin‐V+7‐AAD+	*p* Value	Annexin‐V–7‐AAD+	*p* Value
**–**	74.1 ± 2.6		19.2 ± 1.0		5.8 ± 1.6		0.8 ± 0.1	
10	73.4 ± 4.1		20.0 ± 3.6		5.9 ± 1.1		0.7 ± 0.1	
15	62.8 ± 4.4		25.6 ± 1.1	0.0015	10.6 ± 4.9		0.9 ± 0.4	
30	35.9 ± 4.2	0.0184	50.3 ± 6.1	0.0417	12.7 ± 3.1		1.1 ± 0.2	

Percent of gated single cells (mean values ± SD), *p* values show significance compared with control using Dunett's multiple comparison test (only values < 0.05 are shown).

7‐AAD, 7‐actinomycin D; CBD, cannabidiol; SD, standard deviation.

### CBD Elevates Intracellular Ca^2+^ in C28/I2 and Primary Human Chondrocytes

**Figure 2:**
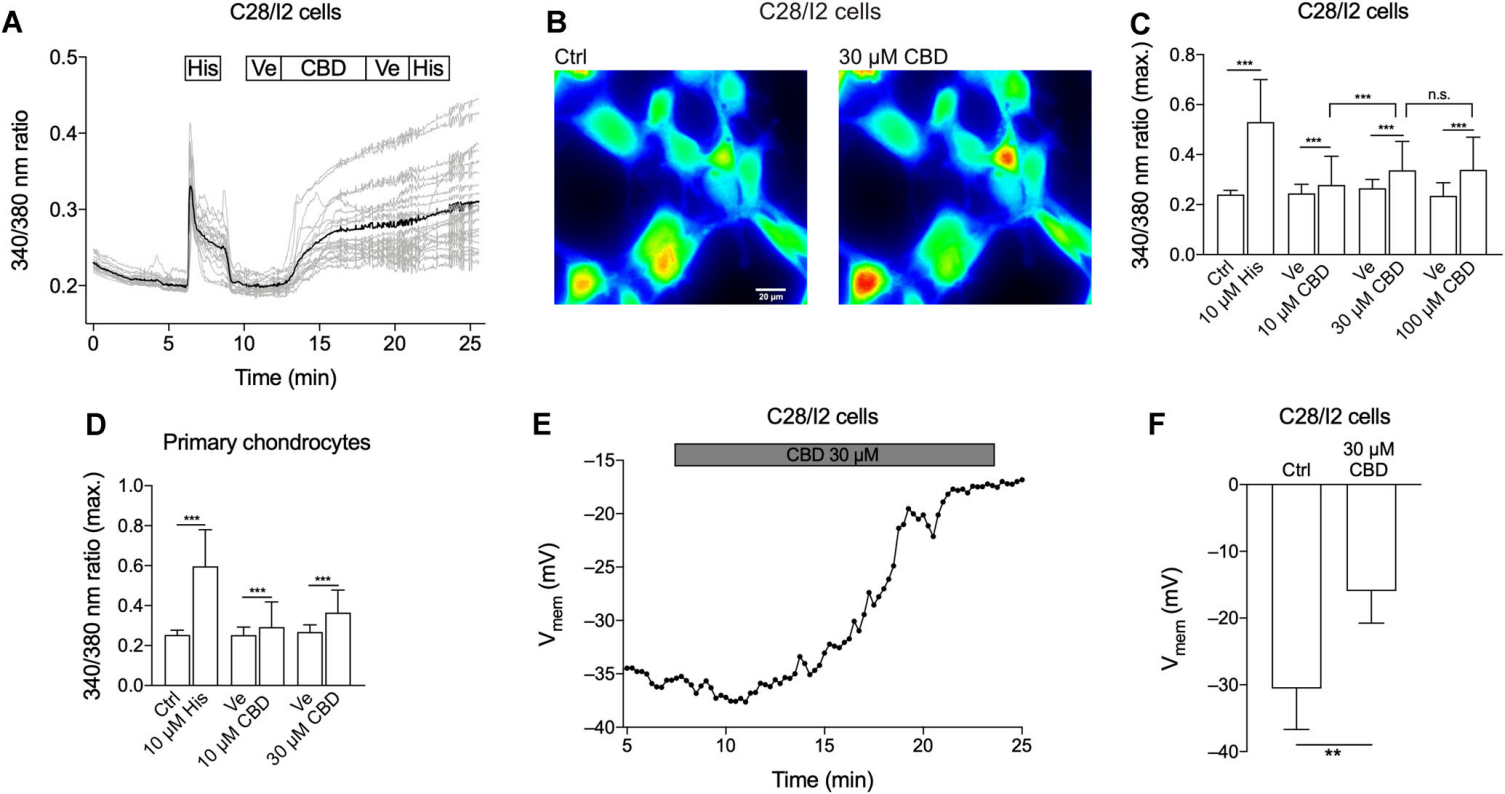
Cannabidiol (CBD) induces [Ca^2+^]*_i_* elevation and a depolarization of the chondrocyte cell membrane potential (*V*_mem_). Time‐lapse fluorescence imaging of [Ca^2+^]*_i_* (A–D) and whole‐cell patch‐clamp time course of *V*_mem_ and mean *V*_mem_ (E, F). (A) Time course of a single [Ca^2+^]*_i_* measurement using Fura‐2/AM. C28/I2 cells were perfused with 10 µM histamine (His) as a positive control, subsequently washed with bath solution containing the CBD solvent, ethanol (Ve = Vehicle) and then treated with 100 µM CBD, followed by another washing step and histamine application. Gray lines represent recordings of single cells, the black line represents the average of the single‐cell recordings. Boxes above indicate the perfusion conditions. (B) Pseudo‐color image of C28/I2 cells stained with Fura‐2/AM. A shift toward yellow and red indicates accumulation of [Ca^2+^]*_i_*. (C) Mean ± standard deviation (SD) of maxima of 340/380 ratios of C28/I2 cells treated with rising concentrations of CBD (10 µM: three independent experiments, 73 single cells; 30 µM: two independent experiments, 85 single cells; 100 µM: five independent experiments; 93 single cells). (D) Mean ± SD of maxima of 340/380 ratios of primary human chondrocytes treated with histamine as positive control followed by rising concentrations of CBD (10 µM: one experiment, six single cells; 30 µM: two independent experiments, 26 single cells). (E) Time course of *V*_mem_ of a single experiment. Each dot represents the mean *V*_mem_ over 15 s. (F) Mean ± SD *V*_mem_ in the absence (Ctrl) and the presence of 30 µM CBD. [Color figure can be viewed at wileyonlinelibrary.com]

To investigate the impact of CBD on Ca^2+^ homeostasis in human articular chondrocytes, we performed life cell imaging experiments with Fura‐2/AM. In addition, the perforated whole‐cell patch‐clamp technique was used for measuring changes of the cell membrane potential (*V*_mem_). Ten micromolar of histamine was used as a positive control and elicited a typical biphasic elevation in [Ca^2+^]*_i_*. Perfusion with CBD triggered a sustained [Ca^2+^]*_i_* elevation in C28/I2 cells (Fig. 2A, B, and C) and primary articular chondrocytes (Fig. 2D) at 10, 30, and 100 µM. Most cells showed a sustained elevated Ca^2+^ level even after washout and only a few cells responded when histamine was administered after CBD treatment (Fig. 2A). A typical time course of an individual experiment is shown in Figure 2A. Human primary chondrocytes showed an equal response to CBD treatment (Fig. 2D). The observed Ca^2+^ influx was in very good accordance with the depolarization of *V*_mem_ in C28/I2 cells from −30.57 ± 2.50 to −15.94 ± 1.96 mV (*p* = 0.028) after treatment with CBD (Fig. 2E and F). The increase from 30 to 100 µM CBD did not further increase the amplitude of the Ca^2+^ signal in the Fura‐2 experiments, but the number of responding cells. We, therefore, used this concentration in the following experiments.

### Influx of Extracellular Ca^2+^ Induces Apoptosis

**Figure 3:**
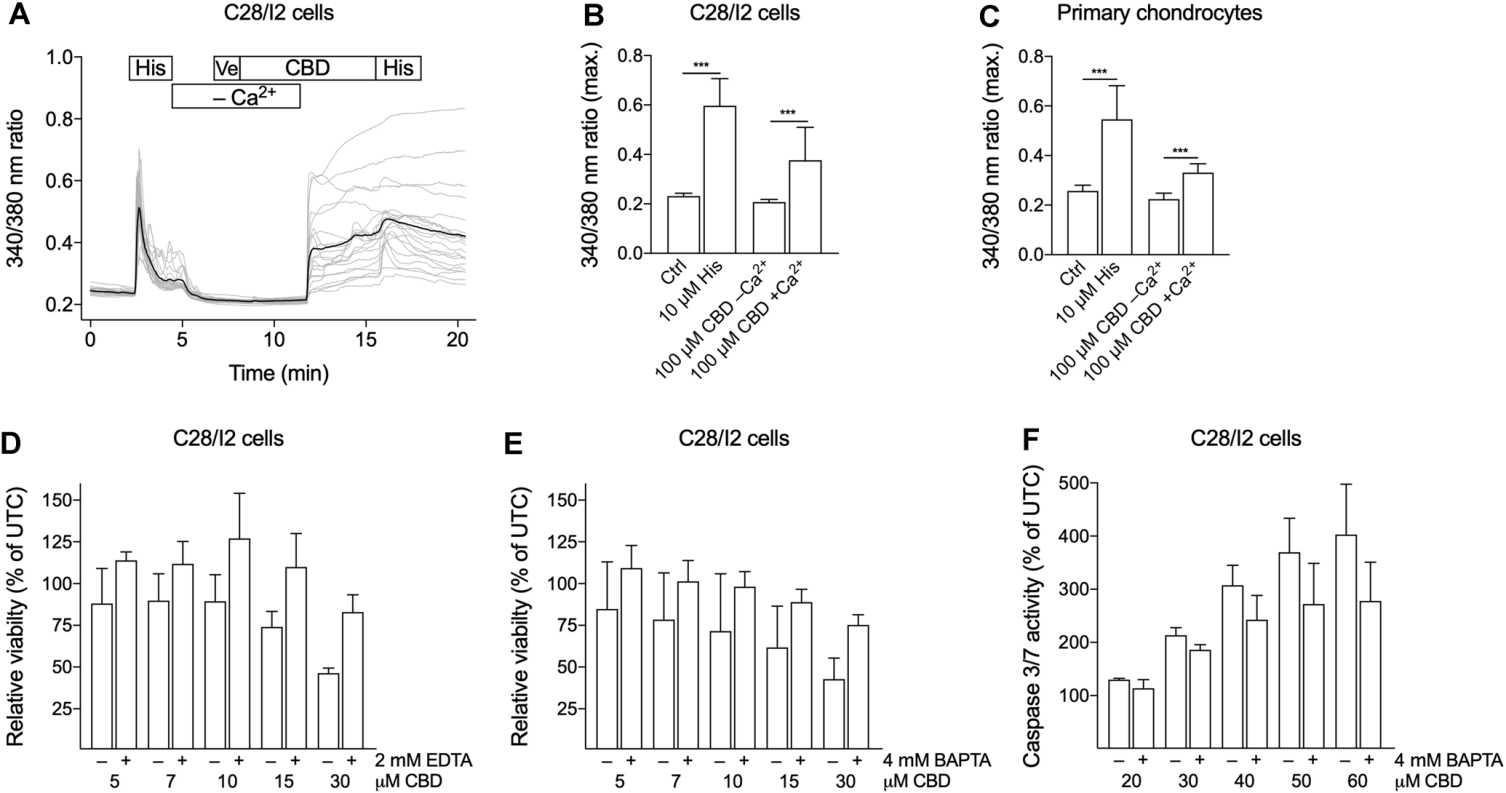
Absence of extracellular Ca^2+^ abolishes the cannabidiol (CBD)‐induced [Ca^2+^]*_i_* elevation and improves cell viability after CBD treatment. Time‐lapse fluorescence imaging of cytosolic Ca^2+^ of C28/I2 and primary chondrocytes with CBD treatment with and without extracellular Ca^2+^ (A–C) and relative viability and caspase 3/7 activity tested in C28/I2 cells with and without Ca^2+^ chelating compounds (D–F). (A) Time course of a single [Ca^2+^]*_i_* measurement. C28/I2 cells were perfused with 10 µM histamine (His) as positive control, subsequently vehicle and CBD without Ca^2+^ were applied, followed by CBD and histamine in the presence of Ca^2+^. (B, C) Mean ± standard deviation (SD) of maxima of 340/380 ratios of C28/I2 cells (B; four independent experiments; 83 single cells) and primary chondrocytes (C; two independent experiments; 55 single cells) treated with 100 µM CBD in the absence or presence of extracellular Ca^2+^. (D, E) Relative cell viability of C28/I2 cells treated with rising concentrations of CBD with or without 2 mM ethylenediaminetetraacetic acid (EDTA) (d) or 4 mM 1,2‐bis(o‐aminophenoxy)ethane‐*N*,*N*,*N*′,*N*′‐tetraacetic acid (BAPTA) (E). (F) Relative caspase 3/7 activity in C28/I2 cells treated with rising concentrations of CBD in the absence or presence of 4 mM BAPTA. (D–F) Means ± SD of three independent experiments.

To establish the source of this [Ca^2+^]*_i_* elevation by CBD, we performed Fura‐2 assays with Ca^2+^‐free bath solutions. In the absence of extracellular Ca^2+^, CBD did not induce an increase in the Fura‐2 ratio, indicating that either Ca^2+^ influx per se or a secondary release from intracellular stores underlies the CBD effect (Fig. 3A–C). Subsequent addition of Ca^2+^ to the bath solution again elicited an increase of the Fura‐2 ratio in C28/I2 cells (Fig. 3A and B) and in primary chondrocytes (Fig. 3C).

In the next step, we investigated, whether the Ca^2+^ influx is linked to the apoptotic effect of CBD. When C28/I2 chondrocytes were co‐treated with the Ca^2+^ chelating compounds EDTA (2 mM) or cell‐permeable BAPTA/AM (4 mM) and rising concentrations of CBD (5–30 µM) for 2 h, relative cell viability was enhanced compared with cells treated with CBD alone (Fig. 3D and E). In addition, caspase 3/7 activity was reduced in BAPTA/AM and CBD co‐treated samples (Fig. 3F). Completely restored cell viability or exhaustive inhibition of caspase 3/7 activity could not be achieved, because the cells did not tolerate complete Ca^2+^ depletion with higher concentrations of chelators for longer exposure times.

### CBD‐Dependent Ca^2+^ Influx is Not Inhibited by Ca^2+^ Channel Blockers CdCl_2_ or Nifedipine

**Figure 4:**
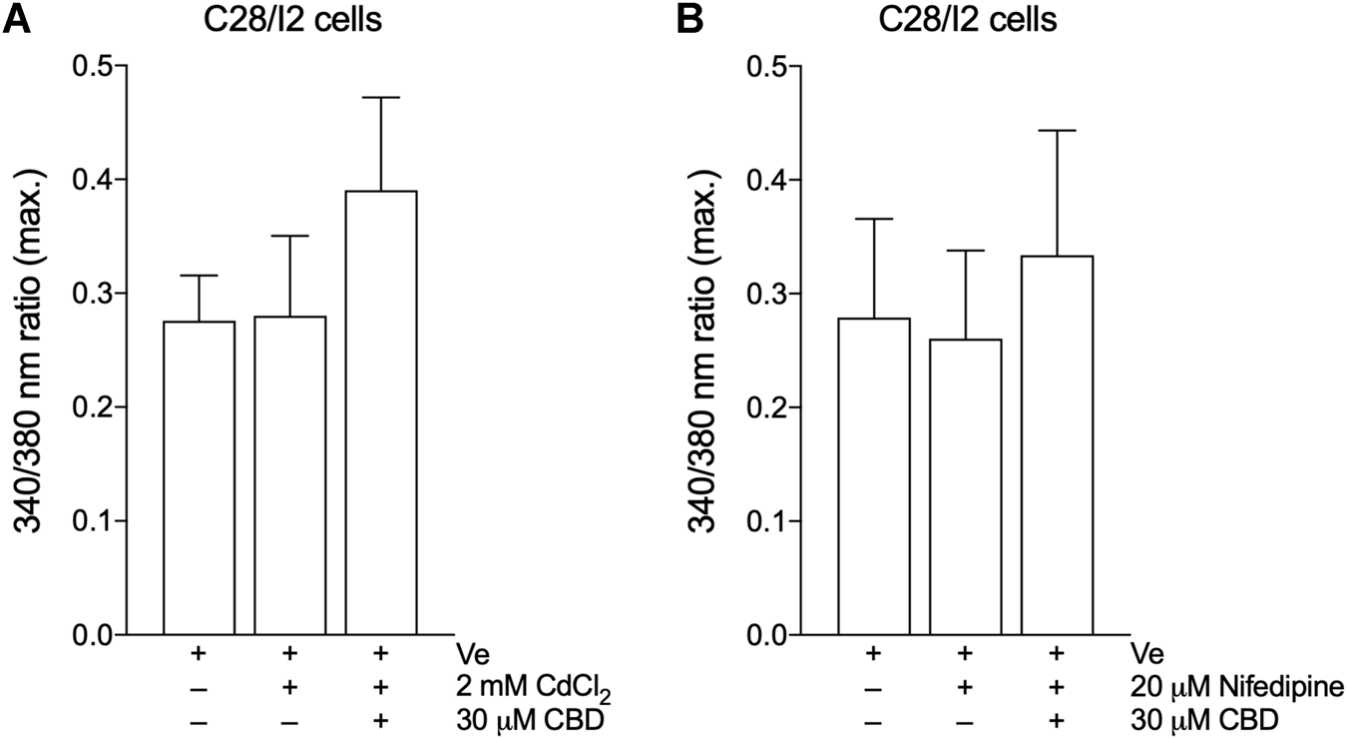
Mean ± standard deviation (SD) of maxima of 340/380 ratios of three independent [Ca^2+^]*_i_* measurements on C28/I2 cells treated with 30 µM CBD in the absence or presence of 2 mM CdCl_2_ (A; three independent experiments; 51 single cells) or 20 µM Nifedipine (B; three independent experiments; 79 single cells).

To further elucidate the pathway of CBD‐induced Ca^2+^ entry, C28/I2 chondrocytes were exposed to Ca^2+^ channel inhibitors prior to CBD treatment. Preceding treatment with the non‐specific Ca^2+^ channel blocker CdCl_2_ (Fig. 4A) or Nifedipine (Fig. 4B), a blocker of voltage‐gated L‐type Ca^2+^ channels, still evoked a rise in the Fura‐2 ratio after addition of CBD. This response was comparable to the treatment with CBD alone (Fig. 1A), indicating that Ca^2+^ channels are not the primary mediators for the [Ca^2+^]*_i_* elevation.

### CBD‐Dependent Ca^2+^ Influx and Apoptosis Are Partially Mediated Via CB1 Receptors

**Figure 5:**
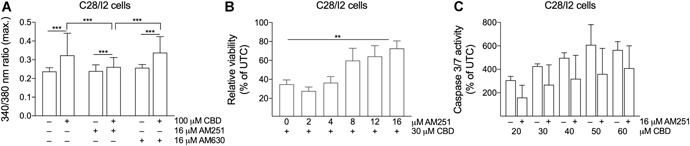
The cannabinoid receptor 1 (CB1) receptor blocker AM251 partially antagonizes cannabidiol (CBD)‐induced effects. (A) Mean ± standard deviation (SD) of maxima of 340/380 ratios of three independent experiments on C28/I2 cells treated with 100 µM CBD in the absence or presence of 16 µM CB1 receptor antagonist AM251 or CB2 receptor antagonist AM630. (B) Relative viability of C28/I2 cells treated with 30 µM CBD and rising concentrations of AM251. (C) Relative caspase 3/7 activity in C28/I2 cells treated with rising concentrations of CBD with or without AM251. Means ± SD of three independent experiments.

Next, we investigated, via Fura‐2 imaging, whether the Ca^2+^ influx is mediated by the classical cannabinoid receptors CB1 or CB2. C28/I2 cells were pre‐treated with the CB1 receptor‐selective antagonist AM251 or the CB2 receptor‐selective antagonist AM630 (16 µM, 2 h) followed by co‐treatment of cells with CBD (100 µM). In AM251 treated cells, the amplitude of the CBD induced [Ca^2+^]*_i_* rise was reduced compared with cells treated solely by CBD. In contrast, treatment with AM630 did not alter the Ca^2+^ response (Fig. 5A). Ca^2+^ entry was not completely abolished after CB1 blocker treatment and still elevated compared with CBD‐untreated cells, indicating the concomitance of additional mechanisms for the entry.

We also explored the influence of CB1 receptor inhibition on cell viability and caspase 3/7 activation. When C28/I2 chondrocytes were pre‐treated with rising concentrations of AM251 (2–16 µM, 2 h), followed by co‐treatment with 30 µm CBD (5 h), cell viability was restored to almost 80 % compared with reduced viability in CBD‐only treated cells (34 %) (Fig. 5B). In parallel, dose‐dependent caspase 3/7 activation under increasing concentrations of CBD could be reduced by treatment with 16 µM AM251 (Fig. 5C).

### CBD‐Enhanced Erk1/2 Phosphorylation is Not Mediated Via CB1

**Figure 6:**
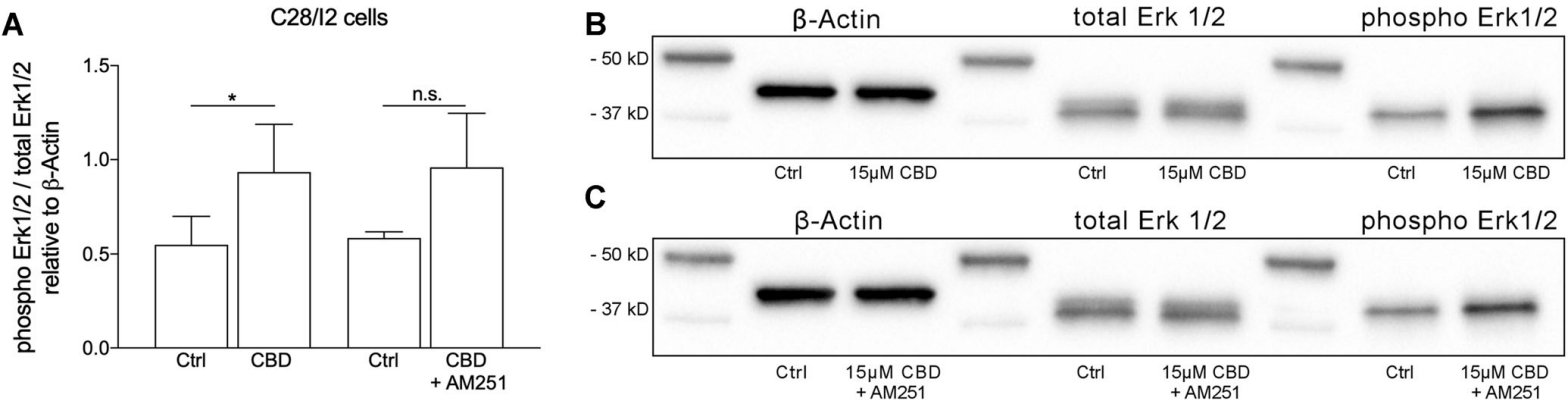
Western blot analysis of Erk1/2 phosphorylation in C28/I2 cells treated with cannabidiol (CBD) (15 µM, 3 h; six independent experiments) in the presence or absence of AM251 (16 µM, 2 h pre‐treatment, 3 h co‐treatment; three independent experiments). (A) Mean ± SD of normalized densitometry values of 6 (CBD) or 3 (CBD + AM251) independent western blot experiments for CBD. (B) and (C) Representative western blots for β‐actin, total Erk1/2, and phospho Erk1/2 under control conditions (Ctrl), in the presence of CBD and after co‐treatment with CBD and AM251.

Levels of total and phosphorylated Erk1/2 were quantified in lysates of CBD treated (15 µM, 3 h) C28/I2 cells by Western blotting. CBD induced an increase in phosphorylation of Erk1/2 compared to untreated controls (Fig. 6A and B). Co‐treatment with CB1 receptor blocker AM251 did not affect this phosphorylation (Fig. 6C), again implicating the involvement of additional signaling pathways other than CB1 activation in the actions of CBD.

## DISCUSSION

To the best of our knowledge, the present study is the first to investigate the effects of the non‐psychoactive phytocannabinoid CBD on human articular chondrocytes. Regardless of intensive research during the last few decades, the molecular basis of the pharmacological effects of CBD remains elusive. On the one hand, neuroprotective and pro‐survival effects have been shown in several cell types^23^, ^24^, ^25^; on the other hand, growing evidence has accumulated that CBD can exert anti‐proliferative and pro‐apoptotic effects on a variety of cancer cell types like glioma cells, lung, breast, and prostate cancer cells.^26^, ^27^, ^28^, ^29^ In the context of OA, only a few studies report on anti‐inflammatory and analgesic effects of CBD in animal models of arthritis. Chondrocytes are the unique cellular residents of articular cartilage and are responsible for the maintenance of extracellular matrix homeostasis. There is a strong correlation between cartilage degeneration and chondrocyte apoptosis in the pathogenesis of OA. So far, no scientific evidence for effects of CBD on chondrocytes or cartilage metabolism has been provided. Therefore, we first conducted experiments investigating cell viability and apoptosis in human articular chondrocytes after CBD treatment. We were able to show that CBD at micromolar concentrations reduces cell viability both in a chondrocyte cell line as well as primary chondrocytes. This viability reduction was accompanied by a dose‐dependent increase of early apoptotic cells and caspase 3/7 activity in the C28/I2 cell line.

As cannabinoids have been shown to promote changes in Ca^2+^ homeostasis,^30^, ^31^, ^32^ we investigated the influence of CBD treatment on [Ca^2+^]*_i_* levels in human articular chondrocytes. We were able to demonstrate a sustained elevation of [Ca^2+^]*_i_* after CBD treatment in live‐cell imaging experiments. This increase was absent after removal of extracellular Ca^2+^. Moreover, we could link the Ca^2+^ influx to the apoptotic actions of CBD in chondrocytes by showing a reduction of apoptosis when Ca^2+^ chelating compounds were co‐applied with CBD. These findings are partially in disagreement with the study of Drysdale et al.,^33^ who described a very similar [Ca^2+^]*_i_* increase in hippocampal cells after administration of CBD, but, in contrast, found no reduction of cell viability after incubation with CBD (1 and 10 µM) for 48 h. Dysregulation of Ca^2+^ homeostasis and subsequent apoptosis induction by CBD application has on the other hand been verified for rat oligodendrocytes, human breast carcinoma cell lines, human glioma cell lines and leukemia cells.^34^, ^35^, ^36^ As aforementioned, there is a lack of studies concerning the actions of CBD on chondrocytes. We could only find one study concerning the effect of the EC AEA on murine and human chondrocytes, which supports our findings by demonstrating the lethal effect of AEA. In contrast, protective effects of CBD have been shown among others for nucleus pulposus cells treated with H_2_O_2_ and CBD or oligodendrocyte cells.^23^, ^24^ These contradicting results suggest a very specific and cell‐type‐dependent action of CBD on cell viability, whereupon, our findings implicate a potential harmful role of CBD for chondrocytes. Interestingly, primary chondrocytes showed enhanced tolerance to CBD treatment at low concentrations compared to the immortalized cell line, indicating a possible difference of CBD effects between primary cells and immortalized or cancer cells.

To target the mediators of CBD action in chondrocytes, we performed a series of experiments with different blocking agents. Neither unspecific Ca^2+^ channels inhibition by CdCl_2_ nor a specific blockage of L‐type voltage‐gated channels by Nifedipine reduced the CBD‐induced Ca^2+^ entry. Given the high blocker concentrations used, we assume that the ineffectiveness of the inhibitors to counteract Ca^2+^ entry is not due to an override of the blocker action by high CBD concentrations. CBD binds with low affinity to CB1 and CB2 receptors. These receptors are considered not to be directly targeted by CBD, but other studies have confirmed indirect CBD‐mediated agonism and antagonism of the CB1 receptor.^37^, ^38^ Both the CB1 and CB2 receptor are expressed in human articular cartilage as well as the C28/I2 cell line,^14^, ^18^ and we, therefore, investigated whether the CBD‐induced Ca^2+^ influx is influenced by blockage of those receptors. We were able to demonstrate a decrease of the CBD‐evoked Ca^2+^ amplitude in Fura‐2 assays by specifically blocking the CB1 receptor, whereas blocking the CB2 receptor left the signal unaffected. Also, the apoptotic effects were at least partially mediated by the CB1 receptor, as receptor blockage improved cell viability and reduced caspase activity.

CB1 receptors exert their actions through a variety of signaling pathways including the mitogen‐activated protein kinase (MAPK) pathways.^39^ We were able to demonstrate a CBD induced upregulation of Erk1/2 phosphorylation, which was shown to be CB1‐receptor‐independent. As the Ras/Raf/Erk pathway is involved in apoptosis induction and is upregulated in chondrocytes of OA patients,^40^ the upregulation of phosphorylated Erk1/2 substantiates our findings that CBD at micromolar concentration leads to apoptosis and cell death in chondrocytes.

In summary, we were able to demonstrate that CBD affects Ca^2+^ homeostasis and induces apoptosis in human articular chondrocytes. These effects are partially mediated by the CB1 receptor. Whether this is due to the direct interaction of CBD with the CB1 receptor or indirect mechanisms cannot be deduced from our data. CBD has been shown to interact with a variety of known receptors, for example, TRPV1/2, GPCR55, PPARγ, 5‐HT1A (5‐hydroxytryptamine receptor subtype 1A) and further targets might be identified in the future.^41^ CBD is highly lipophilic and cannabinoids have been shown to interact with cholesterol.^42^, ^43^ Therefore, we cannot exclude unspecific actions via the plasma membrane lipid bilayer, especially at the relatively high concentrations of CBD used throughout our study. Physiological EC plasma concentrations lie in the nanomolar range, but much higher local concentrations during inflammation or other pathological conditions have been proposed.^43^, ^44^ A bell‐shaped dose‐response curve for cannabinoids has often been proposed, and we cannot exclude beneficial effects of CBD at lower concentrations. This will be specifically addressed in an upcoming study. Considering the prevailing discussion about the use of CBD in the treatment of rheumatic diseases and chronic pain manifestations, future studies will be necessary to elucidate the biological mechanisms and downstream signaling pathways underlying the CBD effects.

## ACKNOWLEDGMENT

We thank Dr. Gerhard Nahler and Dr. Eberhard Pirich (Trigal Pharma GmbH) for the generous gift of CBD.

## Supporting information

Supporting information
