# Supplementary material for: Dose‐Dependent Cannabidiol‐Induced Elevation of Intracellular Calcium and Apoptosis in Human Articular Chondrocytes
Source: J Orthop Res. 2019 Aug 26;37(12):2540–9. doi: 10.1002/jor.24430 (PMC6899975; doi:10.1002/jor.24430)
Supplement: Supplementary file 1 — Supporting information [file JOR-37-2540-s001.docx]

**
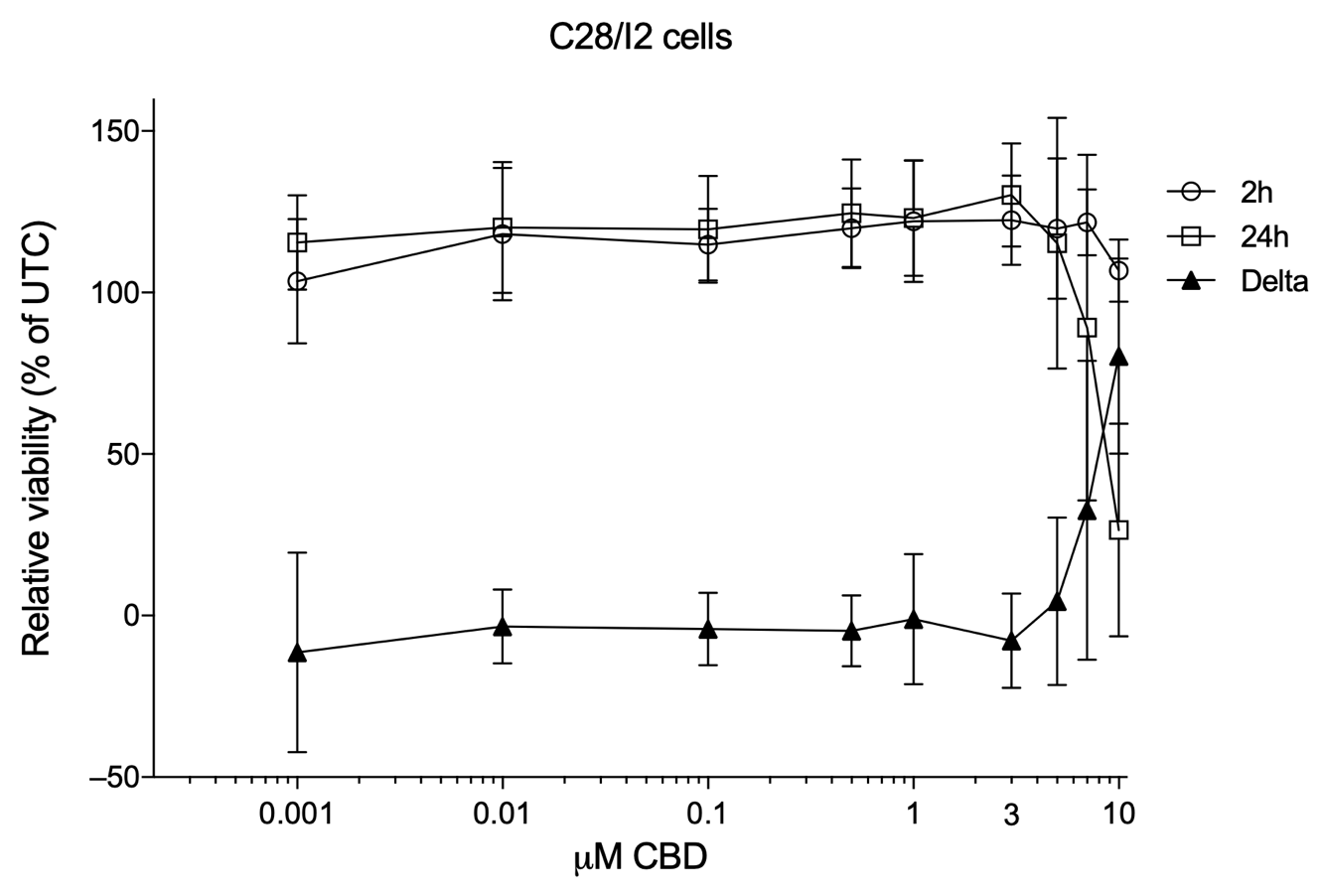
**

**Fig.S1.** Relative viability of C28/I2 cells treated with 0.001 µM to 10 µM CBD for 2 and 24 hours. Results are represented as % of untreated controls (UTC). Mean ± SD of 3 independent Resazurin assays.


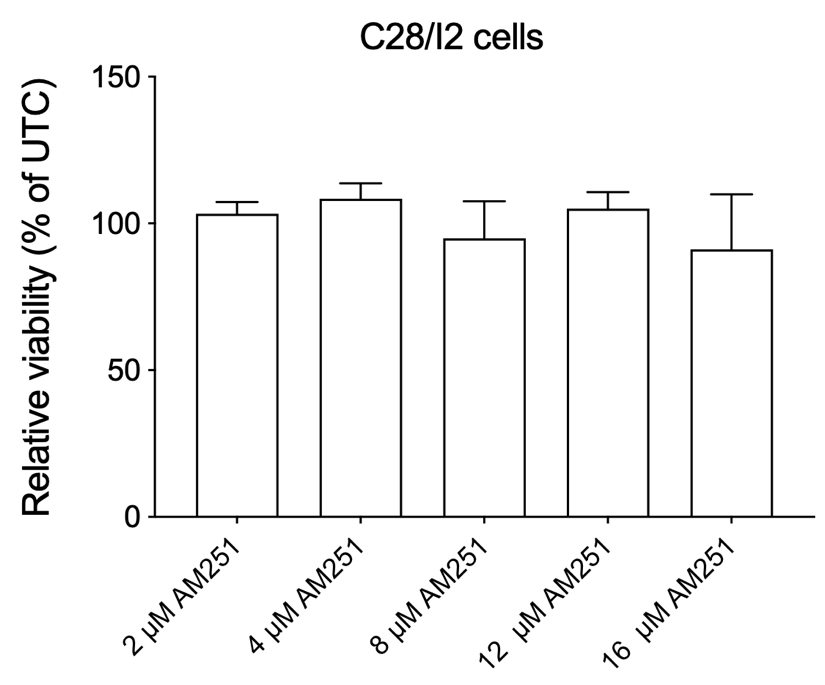


**Fig.S2.** Relative viability of C28/I2 cells treated with rising concentrations of AM251 (2, 4, 8, 12 and 16 µM AM251). Results are represented as % of untreated controls (UTC). Mean ± SD of 3 independent Resazurin assays.
